# The synergy of the XPO1 inhibitors combined with the BET inhibitor INCB057643 in high-grade B-cell lymphoma via downregulation of MYC expression

**DOI:** 10.1038/s41598-023-45721-z

**Published:** 2023-10-29

**Authors:** Manman Deng, Jinshui Tan, Ziying Fan, Lan V. Pham, Feng Zhu, Xiaosheng Fang, Haijun Zhao, Kenh Young, Bing Xu

**Affiliations:** 1 Department of Hematology, The First Affiliated Hospital of Xiamen University and Institute of Hematology, School of Medicine, Xiamen University, Xiamen, 361003 China; 2 Key Laboratory of Xiamen for Diagnosis and Treatment of Hematological Malignancy, Xiamen, 361102 China; 3 Department of Hematology, Dongguan People’s Hospital, Dongguan, 523000 China; 4 Phamacyclics, an Abbvie Company, San Francisco, CA USA; 5 Department of Hematology, The Affiliated Hospital of Xuzhou Medical University, Xuzhou, Jiangsu China; 6 Department of Hematology, Shandong Provincial Hospital Affiliated to Shandong University, Jinan, China; 7 Division of Hematopathology and Department of Pathology, Duke University Medical Center, Durham, NC USA; 8 Department of Hematology, the First Affiliated Hospital of Xiamen University and Institute of Hematology, Medical College of Xiamen University, No.55, Zhenhai Road, Siming District, Xiamen, 361003 Fujian China

**Keywords:** Drug development, Targeted therapies, B-cell lymphoma

## Abstract

High grade B-cell lymphoma with MYC and BCL2 rearrangements (HGBCL-DH) represents an uncommon B-cell lymphoma (BCL) with aggressive clinical courses and poor prognosis. Despite revolutionary therapeutic advances in BCL, there has been limited treatment progress in HGBCL-DH, thus necessitating additional therapeutic strategies for HGBCL-DH. This study demonstrated that the BET antagonist INCB057643 synergized with the XPO1 inhibitors (selinexor and eltanexor) to decrease cell viability and increase cell apoptosis in HGBCL-DH cells with or without TP53 mutations. As anticipated, the combined treatment of INCB057643 with selinexor slowed tumor growth and reduced the tumor burden in TP53-mutated HGBCL-DH xenografts. Mechanistically, MYC functional inhibition was a potential molecular mechanism underlying the synergy of the combined INCB057643 and selinexor treatment in HGBCL-DH cells independent of TP53 mutation status. In TP53 mutated HGBCL-DH cells, inducing DNA damage and impairing the DNA damage response (DDR) were involved in the therapeutic interaction of the combined regimen. In TP53 wild-type cells, the molecular mechanism was linked with upregulation of p53 levels and activation of its targeted pathways, rather than dysregulation of the DDR. Collectively, we might provide a potential promising combination therapy regimen for the management of HGBCL-DH. Clinical evaluations are warranted to confirm this conclusion.

## Introduction

High-grade B-cell lymphoma with MYC and BCL2 rearrangements (HGBCL-DH), historically termed double-hit lymphoma (DHL), accounts for 5–10% of diffuse large B-cell lymphoma (DLBCL) cases, a newly defined lymphoma condition in the 2016 version of the WHO lymphoma classification with a very poor prognosis^1–3^. TP53 mutation is a common event in patients with HGBCL-DH and further worsens their clinical outcomes^4,5^. Despite great progress in the treatment of other aggressive B-cell lymphomas, there are limited treatment options for patients with HGBCL-DH, especially for those with TP53 mutations^6–9^. Therefore, identifying additional effective treatment strategies is an unmet medical need for patients with HGBCL-DH, particularly those with TP53 mutations.

XPO1 is a nuclear exporter responsible for the cytoplasmic localization of hundreds of proteins and mRNAs, including many tumor suppressors and oncogenic factors, such as p53, IκB, p21, and MYC^10–13^. XPO1 is essential for normal physiological functions and has been found to be upregulated in multiple malignant diseases associated with advanced clinical stages, aggressive behaviors and unfavorable prognoses^14–16^. Selinexor and eltanexor, well-known selective inhibitors of nuclear export, show potent inhibitory activities against XPO1-mediated nucleocytoplasmic transport^17–19^. Several clinical trials have reported the impressive antitumor efficacies of selinexor in combination with other drugs in various tumor types^19–21^. However, therapeutic responses to XPO1 inhibitor monotherapy remain unsatisfactory^17,18,22,23^, suggesting that combination treatments are necessary to improve the cytotoxic effects of XPO1 inhibitors against tumor cells. The SADAL study evaluated the antilymphoma effectiveness of selinexor in patients with recurrent DLBCL^18^. Due to its antilymphoma efficacy reported in the SADAL trial, selinexor has been approved by the FDA for the treatment of recurrent DLBCL patients^24^. In this study, selinexor monotherapy yielded an overall response rate of only 28%, with a complete remission rate of 12% among all 127 DLBCL patients. Patients with MYC-overexpressing DLBCL had poorer responses to selinexor than those without MYC overexpression, but no data regarding the clinical responses of this drug in patients with HGBCL-DH have been reported^18^. Our preclinical results had previously revealed that the cytotoxicity of selinexor in HGBCL-DH cells was not inferior to that in non-HGBCL-DH cells^15^. However, our data showed that TP53-mutated HGBCL-DH cells were more resistant to the antitumor effects of selinexor monotherapy than TP53 wild-type HGBCL-DH cells. Notably, our preliminary studies revealed that the novel BET inhibitor INCB057643 potentiated the antilymphoma activities of selinexor in killing HGBCL-DH cells independent of TP53 mutation status^15^.

In the current study, we sought to further confirm the therapeutic interaction of XPO1 inhibitor (including selinexor and eltanexor) with INCB057643 in HGBCL-DH cells with or without TP53 mutations in vitro and in vivo, and to determine the potential mechanism of action.

## Materials and methods

### Cell culture and reagents

HGBCL-DH cell lines with wild-type TP53 (OCI-LY19, RC, Pfeiffer, and WILL2) or mutated TP53 (TMD8, VAL, FN, HF and Toledo) were kindly provided by Drs. Pham and Ford from the MD Anderson Cancer Center Laboratory^5,15^. The details of these cell lines have been published elsewhere. HGBCL-DH cells were cultured in RPMI-1640 medium (Gibco, Life Technologies, NY, USA) supplemented with 10% fetal bovine serum (FBS; Gibco, USA) with 100 units/mL penicillin and 100 μg/mL streptomycin in a 5% CO2 incubator. Selinexor, eltanexor and INCB057643 were purchased from Selleck (Texas, USA).

### Cell viability assay

The HGBCL cell lines were separately plated in 384-well plates at a density of 5000–10,000 cells per well and were then treated with selinexor and INCB057643 alone or in combination for 72 h. Subsequently, cell viability was determined with a CellTiter-Glo Luminescent Cell Viability Assay following the manufacturer’s instructions (Promega, Madison, WI). The assay was performed in biological triplicate with three replicates in each assay.

### Cell apoptosis assay

Drug-treated (XPO1 inhibitor and INCB057643 alone or in combination for 72 h) HGBCL-DH cells were assessed by Annexin V/PI (eBioscience, San Diego, USA) double staining to evaluate apoptotic cell death. The specific experimental details have been described in our previously published studies^5,15^.

### Xenograft model of TP53-mutated HGBCL-DH

A total of 16 male NOD-Prkdc^−/−^ IL2rg^−/−^ (NPI) mice were purchased from IDMO Ltd. (Beijing, China) and housed in the animal center at Xiamen University in a specific pathogen-free (SPF) environment following animal care guidelines. After pretreatment with total body irradiation (1.5 Gy), each NPI mouse was subcutaneously injected with 1 × 107 TMD8 cells, and the tumor volume was calculated daily. When the subcutaneous tumor volume was ≥ 100 mm^2^, the TMD8 xenografts were randomized to the control (vehicle), selinexor (10 mg/kg, 3 times weekly), INCB057643 (10 mg/kg, once daily) and combination regimen groups for a 2-week treatment course. During the treatment course, tumor volume and mouse body weight were monitored every other day. In addition, we monitored the mice for other adverse effects (including diarrhea, fatigue and drowsiness, etc.) related to drug therapies. Finally, mice in each group were euthanized, and the subcutaneous tumors were excised, weighed and photographed to assess the antilymphoma interaction of selinexor with INCB057643 in this in vivo model.

### Western blot analysis

Protein was extracted from HGBCL-DH cells exposed to different treatments with protein lysis buffer containing protease and phosphatase inhibitors. Subsequently, the cell lysates were subjected to SDS–PAGE on a 4–15% gel, and the proteins were transferred onto PVDF membranes. Next, targeted proteins were analyzed with specific primary antibodies and HRP-conjugated secondary antibodies (1:10,000, Cell Signaling Technology). Proteins were visualized using an electrochemiluminescence (ECL) system (Amersham, Little Chalfont, UK). Antibodies against p53, p21, MYC, Survivin, BCL2, MCL1, Puma, Bax, NOXA, BAK, BIM, BCL-XL, MDM2, ATM/p-ATM, ATR/p-ATR, Wee1/p-Wee1, CHK1/p-CHK1, CHK2/p-CHK2, RAD51, γH2AX, and β-actin were obtained from Cell Signaling Technology (Danvers, MA). In the assays, PVDF membranes with transferred proteins were cut horizontally on the basis of molecular weights of targeted proteins. Some close ranges of targeted proteins were stripped and reprobed in the same membrane using different species source of primary antibodies.

### Real-time polymerase chain reaction

TMD8 and OCI-LY19 cells were treated with selinexor (125 nM for OCI-LY19 cells, 500 nM for TMD8 cells) and INCB057643 (2500 nM for OCI-LY19 cells, 1000 nM for TMD8 cells) alone or in combination for 24 h. Total mRNA was separately extracted from TMD8 and OCI-LY19 cells treated as described above and reverse transcribed into cDNA using Evo M-MLV RT Premix for qPCR (Cat No. AG11706, Hunan, China). The expression of the targeted genes was evaluated with a SYBR® Green Premix Pro Taq HS qPCR Kit II (Cat No. AG11702, Hunan, China) according to the user’s manual. The primer sequences were as follows:

β-actin: forward primer, AGGTGTGCACCTTTTATTGGTCTCAA.

reverse Primer, TGTATGAAGGTTTGGTCTCCCT.

c-Myc: forward primer, GCTCATTTCTGAAGAGGACTTGT.

reverse Primer, AGGCAGTTTACATTATGGCTAAATC.

β-Actin was used as the housekeeping control.

### TUNEL fluorescence assay

Subcutaneous tissue sections were deparaffinized by two incubations with xylene for 20 min each followed by dehydration in an ethanol gradient of 100%, 95%, 90%, 80%, and 70%. For antigen retrieval, proteinase K solution was added to the dehydrated sections for 25 min at 37 °C, and the sections were then permeabilized. The prepared sections were subsequently incubated with a TDT enzyme-containing TUNEL reaction solution for 2 h at 37 °C, and nuclei were then counterstained with DAPI. Sections were coverslipped with antifade mounting medium, and fluorescence images were acquired with a fluorescence microscope (Zeiss).

### Statistical analysis

The values are presented as the mean ± standard error of the mean (SEM) of at least three independent experiments. All statistical analyses were conducted with GraphPad Prism 8.0 software. Two-tailed Student’s t test was employed to compare differences between two groups, while one-way analysis of variance (ANOVA) followed by the Bonferroni post hoc test was used for comparisons between more than two groups. P < 0.05 was considered statistically significant.

### Methods statements

All methods including those used in animal models are in accordance with the relevant guidelines and regulations. For example, the Xenograft model of TP53-mutated HGBCL-DH was established and treated with distinct treatment groups according to the ARRIVE guidelines 2.0. We ensure that all gels/blots used in figures comply with the digital image and integrity policies.

### Institutional review board statement

The animal study protocol was approved by the Laboratory Animal Ethics and Management Committee of Xiamen University (XMULAC20170065).

## Results

### Co-inhibition of XPO1 and BET roles synergistically reduces viability and promotes apoptosis in TP53-mutated HGBCL-DH cells

**Figure 1:**
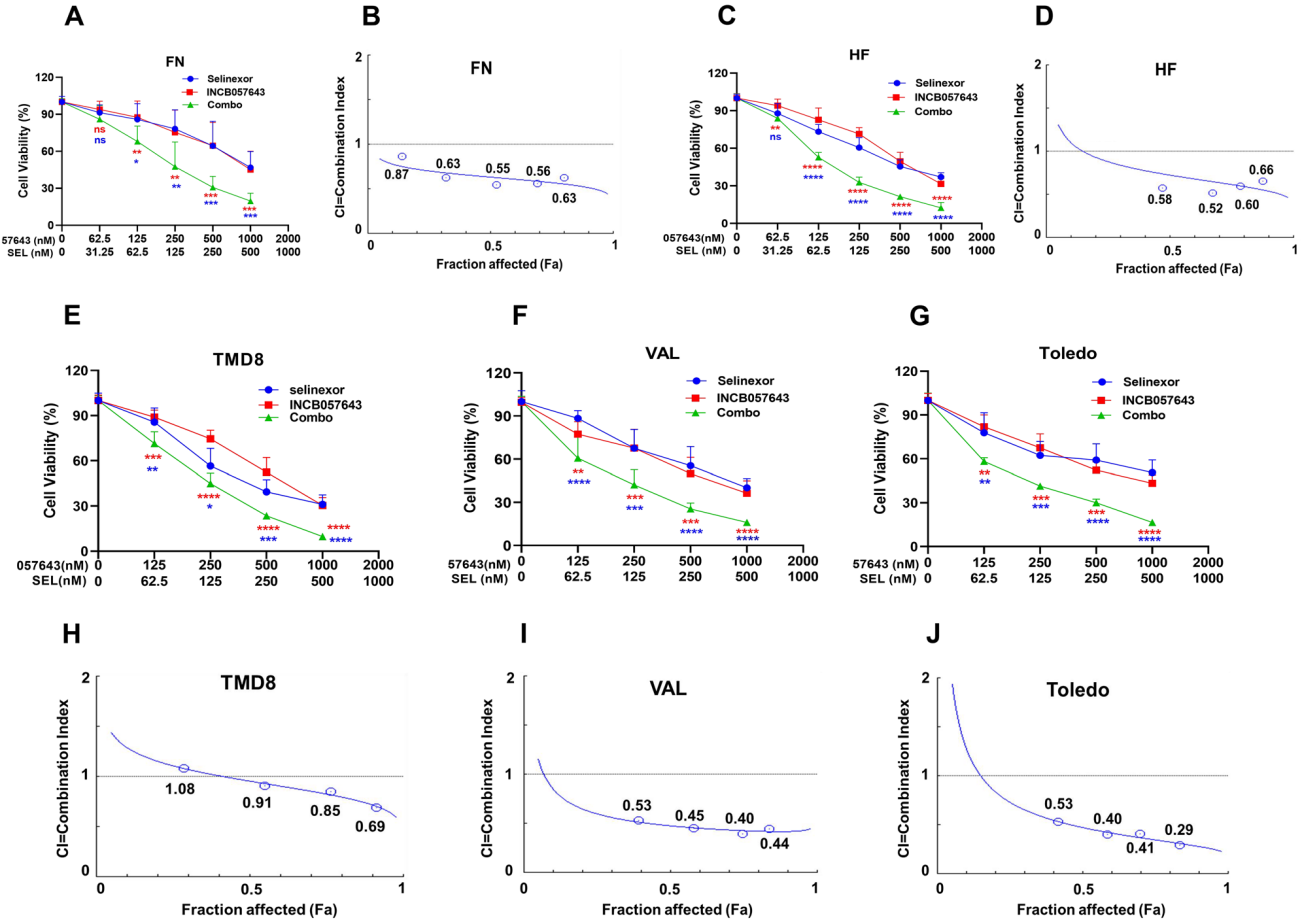
INCB057643 cooperates with selinexor to decrease the viability of HGBCL-DH cells with TP53 mutations. (**A**) INCB057643 enhanced the effects of selinexor on reducing the viability of FN cells. (**B**) Analysis of the CI of selinexor and INCB057643 in FN cells. CompuSyn software was used to calculate the CI based on the Chou-Talalay method. CI < 1 indicates a synergistic effect; CI = 1, an additive effect; and CI < 1, an antagonistic effect. (**C**) Assessment of the viability of HF cells treated with INCB057643 and selinexor alone or in combination for 72 h. (**D**) Calculated CI values of the two drugs in HF cells as described in (**B**). (**E**–**G**) TMD8, VAL and Toledo cells were exposed to INCB057643 and selinexor alone or in combination for 72 h, and a cell viability assay was then performed. (**H**–**J**) Selinexor and INCB057643 synergistically decreased the viability of TMD8, VAL and Toledo cells, as evidenced by CI values of < 1. Sel, 057643 and Combo represent selinexor, INCB057643 and their combination, respectively.

BET transcriptionally modulates MYC expression, and pharmacological inhibition of BET function thus results in MYC downregulation^25^. Numerous BET inhibitors, including INCB057643, have been evaluated in clinical trials, with promising antitumor efficacies indicated by preliminary data analysis^26–28^. In this study, we sought to investigate the therapeutic interaction of INCB057643 in combination with the XPO1 inhibitors (selinexor and eltanexor) on TP53-mutated HGBCL-DH cells. Several TP53-mutated HGBCL-DH cell lines were separately treated with selinexor and INCB057643 alone or their combination at various constant drug concentration ratios for 72 h. Subsequently, cell viability was assessed with a CellTiter-Glo luminescence assay. Compared with either monotherapy, combination treatment with selinexor and INCB057643 led to a significant reduction in viability in the TP53-mutated FN cell line (Fig. 1A). The drug–drug combination index (CI) was calculated with CompuSyn software based on cell viability data and was utilized to assess the therapeutic interaction of the drug combination, with CI < 1 indicating a synergistic effect, CI = 1 indicating an additive effect, and CI > 1 indicating an antagonistic effect. Encouragingly, all CI values of selinexor and INCB057643 combination treatment in FN cells were less than 1 (Fig. 1B), suggesting that selinexor synergizes with INCB057643 to decrease FN cell viability. Moreover, similar therapeutic synergism of the two drugs was confirmed in the remaining abovementioned TP53-mutated cell lines (HF, TMD8, VAL and Toledo) (Fig. 1C–J).

**Figure 2:**
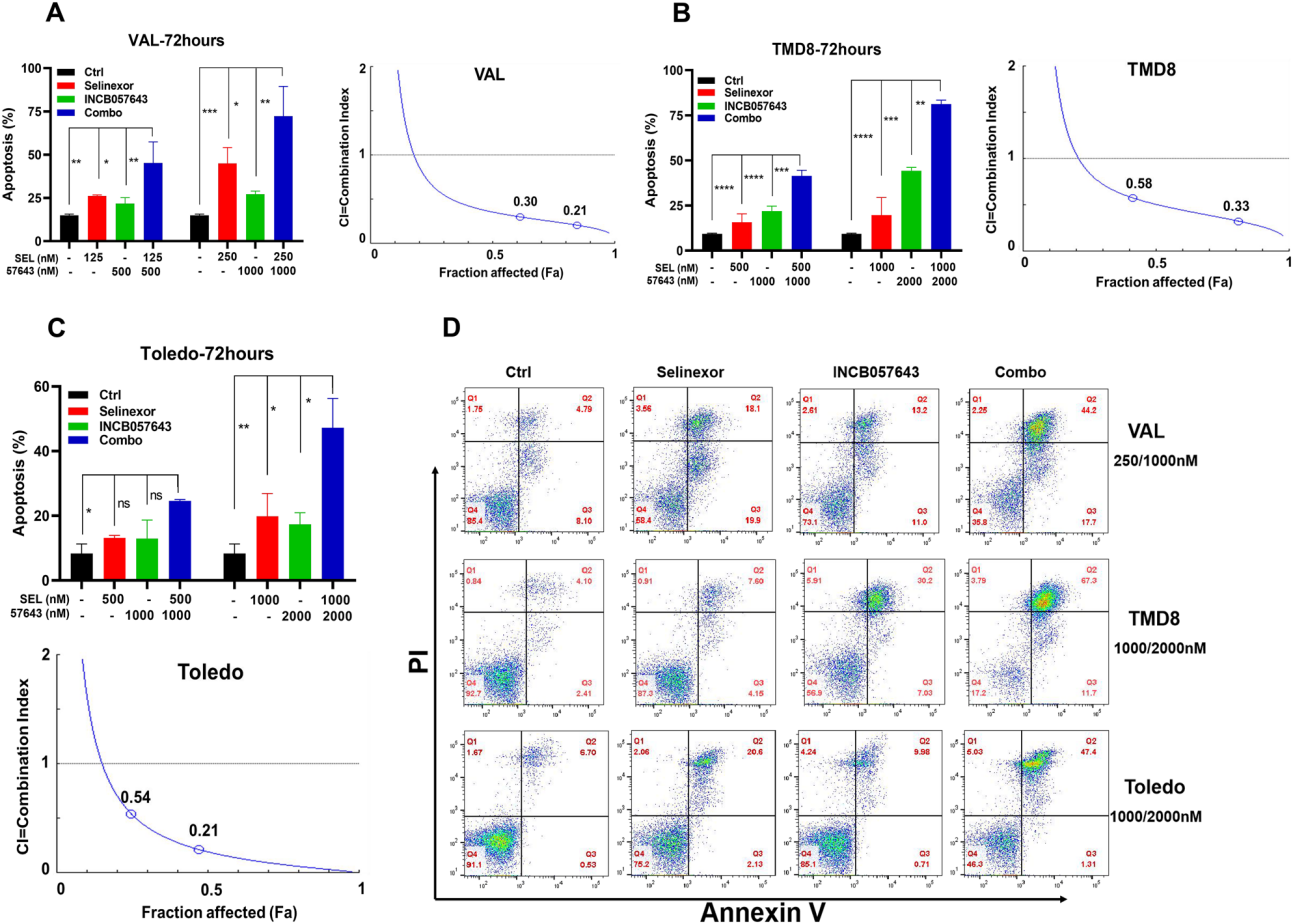
Synergistic proapoptotic effects of INCB057643 combined with selinexor in HGBCL-DH cells with TP53 mutations. (**A**,**B**) INCB057643 synergized with selinexor to promote apoptosis in VAL (**A**) and TMD8 (**B**) cells. The left panel shows the specific apoptosis rates, and the right panel shows the CI values. (**C**) Analysis of the therapeutic interaction of selinexor and INCB057643 in inducing apoptosis in Toledo cells. The top and bottom panels show the specific apoptosis rates and CI values, respectively. (**D**) Representative flow plots of VAL, TMD8 and Toledo cells treated as described in (**A**–**C**).

Next, an Annexin V/PI double staining assay was performed to examine apoptosis in TP53-mutated cells treated with XPO1 inhibitor (either selinexor or eltanexor) and INCB057643 alone or in combination for 72 h. Both XPO1 inhibitor (either selinexor or eltanexor) and INCB057643 alone was able to induce apoptosis in VAL, TMD8 and Toledo cells, but the combination of the two drugs had a more potent ability to increase apoptotic death in these cells (Fig. 2A–D, Supplementary Fig. S1A,B). All CI values of selinexor in combination with INCB057643 were < 1 (Fig. 2A–D, Supplementary Fig. S1C), indicating that these drugs synergistically exert proapoptotic effects on the three tested TP53-mutated cell lines. Taken together, our findings reveal that XPO1 inhibitors synergize with INCB057643 to promote cell death in TP53-mutated HGBCL-DH cells.

### Cooperative cytotoxicity of XPO1 inhibitors and INCB057643 in TP53 wild-type HGBCL-DH cells

**Figure 3:**
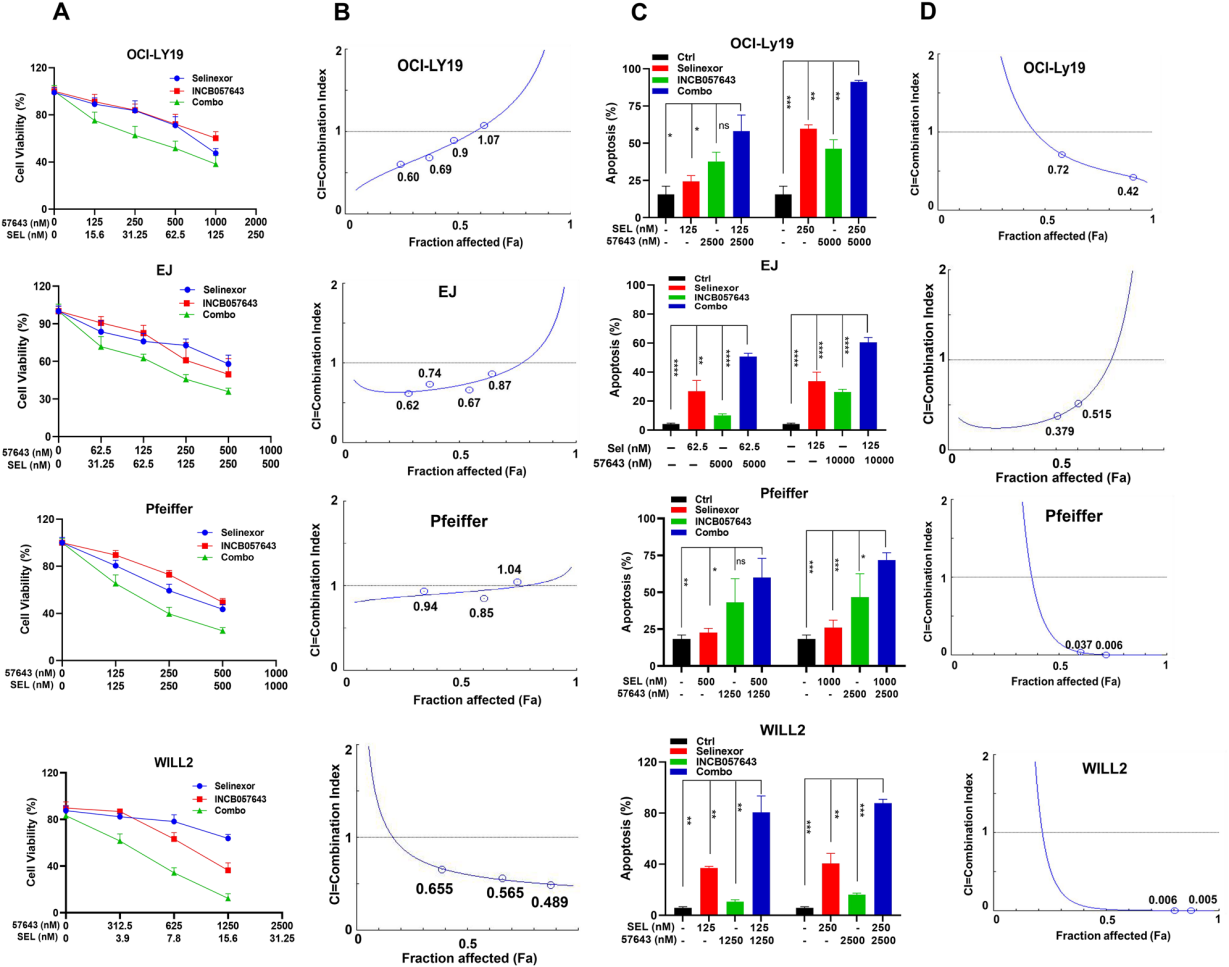
INCB057643 and selinexor synergize to increase HGBCL-DH with wild-type TP53 cell death. (**A**) Analysis of the viability of OCI-LY19, EJ and Pfeiffer cells exposed to INCB057643 and selinexor alone or in combination for 72 h. (**B**) The CI values indicate that INCB057643 and selinexor acted synergistically to reduce the viability of OCI-LY19, EJ and Pfeiffer cells. (**C**) Compared with either monotherapy, INCB057643 and selinexor synergistically enhanced apoptosis in OCI-LY19, EJ and Pfeiffer cells. (**D**) Analysis of the CI values of INCB057643 combined with selinexor in OCI-LY19, EJ and Pfeiffer cells.

To investigate whether XPO1 inhibitors in combination with NCB057643 exert synergistic cytotoxic effects on TP53 wild-type cells, we performed cell viability and apoptosis assays in three TP53 wild-type cell lines treated with XPO1 inhibitors and INCB057643 alone or in combination. OCI-LY19, EJ and Pfeiffer cells were exposed to designated concentrations of selinexor and INCB057643 alone or in combination for 72 h and were then subjected to a CellTiter-Glo luminescence assay. As shown in Fig. 3A (upper panel), the combined treatment had a greater ability to attenuate OCI-LY19 cell viability than that of either agent alone. The CI values indicated that selinexor synergized with INCB057643 in reducing OCI-LY19 cell viability, as demonstrated by the CI of < 1 (Fig. 3B, upper panels). This therapeutic synergy of selinexor coupled with INCB057643 was also observed in the other two TP53 wild-type HGBCL-DH cell lines (Fig. 3A,B, middle and bottom panels). As anticipated, synergistic proapoptotic effects of XPO1 inhibitors combined with INCB057643 were found in the three tested TP53 wild-type cell lines (Fig. 3C,D). Collectively, these results demonstrate that XPO1 inhibitors and INCB057643 exert synergistic cytotoxic effects on TP53 wild-type HGBCL-DH cells.

### In vivo therapeutic interaction of selinexor and INCB057643 in a xenograft model of TP53-mutated HGBCL-DH

**Figure 4:**
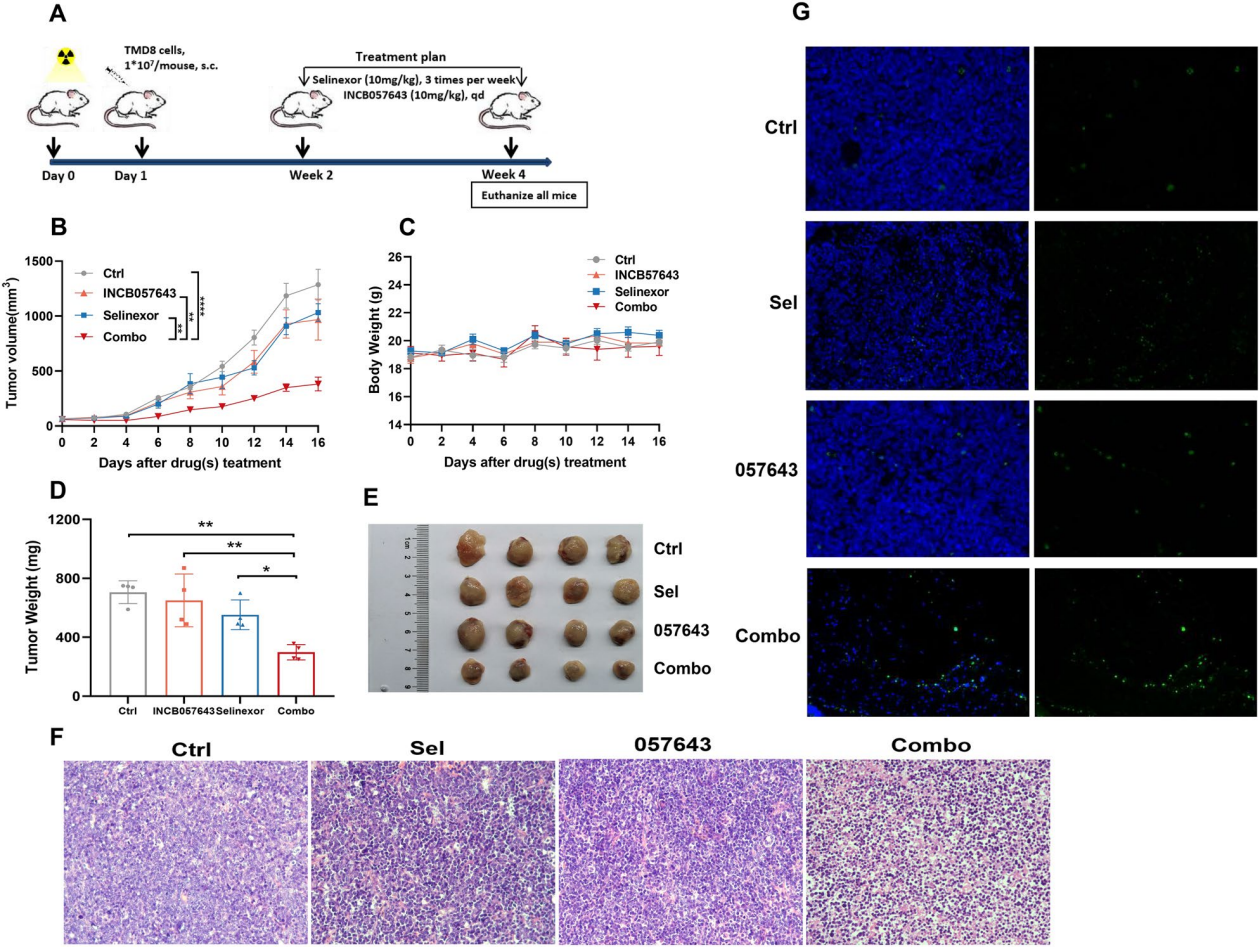
In vivo therapeutic interaction of selinexor and INCB057643 in a xenograft model of TP53-mutated HGBCL-DH. (**A**) Schematic showing the in vivo treatment plan. (**B**) Subcutaneous tumor volumes in the different treatment groups were recorded every two days throughout the treatment course. (**C**) Mouse body weights were monitored every other day. (**D**,**E**) Demonstration of tumor weights (left panel, **D**) and sizes (right panel, **E**) in the vehicle, selinexor, INCB057643 and combination regimen groups. (**F**) Assessment of tumor burden in distinct treatment groups using HE staining. (**G**) The TUNEL assay showed that the combination of selinexor with INCB057643 resulted in a higher apoptosis rate than that of either drug alone. *P < 0.05; **P < 0.01; ****P < 0.0001.

We sought to validate the therapeutic synergism of the combination regimen in vivo by establishing a subcutaneous TP53-mutated TMD8 cell-derived xenograft model of HGBCL-DH. A schematic of the overall plan is provided in Fig. 4A. Selinexor (10 mg/kg, 3 times weekly) and INCB057643 (10 mg/kg, qd) were administered orally alone or in combination over a total of 2 weeks. During the treatment course, subcutaneous tumor volumes and mouse weights were measured and recorded every other day. Consistent with our in vitro findings, selinexor and INCB057643 monotherapies resulted in a reduction in TMD8 xenograft tumor volumes compared with those in the control group (Fig. 4B). More importantly, combined administration of the two drugs had a more potent ability to slow in vivo tumor growth than that of either drug alone (Fig. 4B). No body weight loss was observed with selinexor or INCB057643 treatment alone or in combination (Fig. 4C). In addition, no other remarkable and unacceptable adverse effects were observed in either the monotherapy group or the combined group, suggesting that this combination strategy has favorable safety profiles. At the end of the in vivo experimental treatment course, all TMD8 xenograft-bearing mice in the four treatment groups were euthanized, and the subcutaneous tumors were excised, weighed and photographed. Compared to the untreated group, the groups treated with either selinexor or INCB057643 exhibited decreased tumor weights and sizes, while treatment with selinexor in combination with INCB057643 further decreased the subcutaneous TMD8 tumor xenograft burden (Fig. 4D,E). Hematoxylin and eosin (HE) staining of tumor tissues showed the similar phenomenon that selinexor and INCB057643 monotherapies decreased the tumor burden of TMD8-bearing xenografts compared with the untreated group. Notably, the combination of both drugs further enhanced the effect of decreasing tumor burden in the xenograft model of TP53-mutated HGBCL-DH (Fig. 4F). The TUNEL assay demonstrated that the combined treatment induced higher apoptosis rates than those of either monotherapy (Fig. 4G). In summary, the in vivo experimental data substantiate the synergistic antilymphoma efficacy of selinexor with INCB057643 in HGBCL-DH models.

### Analysis of the mechanism underlying the synergy of selinexor with INCB057643 in HGBCL-DH cells with or without TP53 mutations

**Figure 5:**
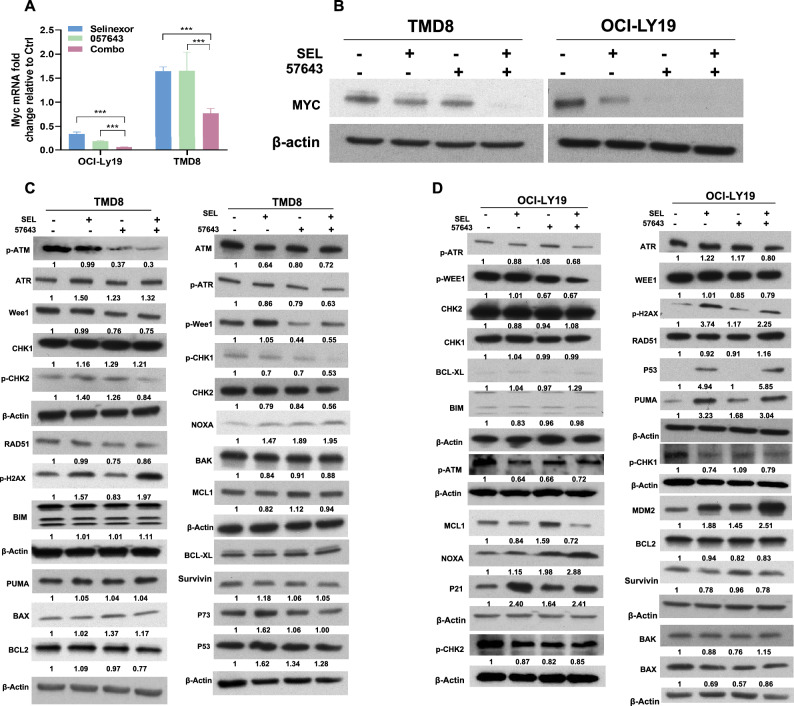
Downregulation of MYC expression contributes to the synergy of INCB057643 and selinexor. (**A**,**B**) qRT–PCR and Western blot analyses revealed that INCB057643 and selinexor interacted with each other to downregulate MYC mRNA (**A**) and protein expression (**B**) in both OCI-LY19 and TMD8 cells. (**C**) Western blot analysis of MYC expression, the potential molecular mechanism of the therapeutic cooperation of INCB057643 and selinexor in TP53-mutated TMD8 cell death. (**D**) Western blot analysis of the indicated proteins in TP53 wild-type OCI-LY19 cells treated with INCB057643 and selinexor alone or in combination. ***P < 0.001.

Since both compounds can decrease MYC expression, we attempted to examine whether downregulation of MYC expression might be involved in the therapeutic cooperation of selinexor and INCB057643. Indeed, compared to either selinexor or INCB057643 alone, the drug combination synergistically decreased MYC expression at both the transcriptional and translational levels in both TP53 wild-type and TP53-mutated HGBCL-DH cells (Fig. 5A,B).

In our previous report, INCB057643 exerted DNA damage-inducing effects on lymphoma cells. Thus, we investigated whether DNA damage and the DNA damage response (DDR) are dysregulated by the combination of these drugs in HGBCL-DH cells. In TP53-mutated cells, compared to treatment with either drug alone, combined treatment with selinexor and INCB057643 markedly elevated the expression level of γH2AX (Fig. 5C), indicating that this drug combination induces DNA damage. Furthermore, the drug combination treatment decreased the phosphorylation levels of several DNA damage repair-related kinases, including ATR, CHK1, CHK2, Wee1 and RAD51, in TP53-mutated TMD8 cells (Fig. 5C). However, inactivation of DNA damage repair-related pathways was not observed in TP53 wild-type OCI-LY19 cells treated with the selinexor/INCB057643 combination (Fig. 5D). In OCI-LY19 cells, selinexor in combination with INCB057643 upregulated the expression of p53 and its downstream targets, including p21, PUMA, MDM2 and Noxa, but did not impact several BCL2 family antiapoptotic (such as BCL2 and BCL-XL) and proapoptotic (such as BAX, BIM, and BAK) mediators (Fig. 5D). In addition, the combination regimen decreased the level of MCL1 but not that of survivin in OCI-LY19 cells. In TMD8 cells, selinexor in combination with INCB057643 did not activate p53-related signaling pathways and did not influence the expression of BCL2 family antiapoptotic and proapoptotic mediators (Fig. 5C). Collectively, these findings indicate that downregulation of MYC expression contributes to the therapeutic synergy of selinexor and INCB057643 in HGBCL-DH cell death, independent of TP53 mutation status.

## Discussion

P53 is a well-known transcription factor involved in many cellular processes, such as apoptosis, cell cycle progression and the DDR^29^. It is considered a tumor suppressor and exerts its antioncogenic effects in the nucleus. TP53 gene mutations usually occur in the DNA-binding domain, and thus, the encoded products of mutant p53 proteins lose their transcription-promoting functions^30,31^. Both wild-type and mutated p53 have two nuclear export signals (NES), one in the N-terminus and one in the C-terminus^32^. Moreover, XPO1 is responsible for the cytoplasmic localization of p53 and its mutants by binding to an NES^12,32,33^. Previous studies have revealed that XPO1 blockade promotes p53 nuclear retention and exerts robust antitumor effects in multiple tumor cell types without TP53 mutations^12^. However, p53-independent signaling pathways play a critical role in the cytotoxicity of selinexor in cancer cell models with or without TP53 mutations^34–36^. In the present study, we observed that the XPO1 inhibitors, including selinexor and eltanexor, resulted in robust antilymphoma effects on HGBCL-DH cells with or without TP53 mutations. The addition of the BET inhibitor INCB057643 synergistically enhanced the antitumoral cytotoxicities of the XPO1 inhibitors on HGBCL-DH cells with or without TP53 mutations. Mechanistically, INCB057643 and selinexor cooperatively downregulated MYC mRNA and protein expression in both TP53-mutated and TP53 wild-type cells, suggesting that a reduction in MYC expression is a potential molecular mechanism of the synergy of this drug combination. In addition to the common mechanism of reducing MYC expression, the combination regimen exhibited therapeutic synergy via different mechanisms of action in TP53-mutated and TP53 wild-type HGBCL-DH cells.

DNA damage constantly occurs due to various endogenous and exogenous genetic insults^37,38^. In response to DNA damage stress, the histone variant H2AX is phosphorylated at its serine 139 residue, forming the well-known DNA damage marker γ-H2AX, and is positioned in the damaged DNA strand to recruit repair kinases. Thus, γ-H2AX formation is considered a characteristic event during DNA damage^39,40^. To cope with DNA damage, organisms have evolved a complicated and coordinated DDR to prevent tumorigenesis^41,42^. The DDR consists of numerous mediators responsible for sensing and responding to different types of DNA damage. An important mediator is ATM, a member of the phosphoinositide-3-kinase-related kinase (PIKK) family. ATM is recruited to sites of DNA damage and then phosphorylates its downstream target kinases, including CHK2 and 53BP1^43^. DNA damage also activates ATR and its target CHK1 to maintain genomic integrity^44^. Rad51 recombinase is a DDR component involved in the repair of DNA strand breaks^45^. Moreover, evidence has revealed that Wee1 deletion by siRNA transfection results in obvious DNA damage induction, indicating that the function of Wee1 is critical to prevent DNA damage^46^. Our proteomic microarray and immunoblot analyses showed that selinexor monotherapy had the ability to increase the level of γ-H2AX in both TP53-mutated and TP53 wild-type HGBCL-DH cells, consistent with the DNA damage-inducing activity of selinexor in acute leukemia cells. Next, we found that the effects of selinexor on γ-H2AX formation were clearly potentiated by combined treatment with INCB057643 in TP53-mutated HGBCL-DH cells but not in TP53 wild-type cells. Studies have revealed that blocking the action of BET with siRNAs or its selective inhibitors, such as JQ1 and INCB057643, can increase DNA damage and impair the DDR in malignant diseases, such as ovarian cancer. Accordingly, our results showed that INCB057643 dephosphorylated several DNA repair proteins, including ATR, CHK1, CHK2, Wee1 and RAD51, in TP53-mutated cells. This impairment of the DDR pathway was further augmented by the combination of selinexor and INCB057643 compared to that of each drug alone. These data demonstrated that enhancement of DNA damage and disruption of DNA damage repair were implicated in the cooperative interaction of this drug combination in TP53-mutated cells.

P21 plays a critical role in controlling cell cycle progression and is a transcriptional target of p53^47^. Both PUMA and NOXA are proapoptotic members of the BCL2 family that are transcriptionally upregulated by the p53 protein^48^. MDM2 is a negative modulator of p53 and is a direct reciprocal downstream target of p53, thus forming a negative feedback loop^49^. These p53-modulated factors are important for sustaining genome stability and preventing oncogenesis. In TP53 wild-type HGBCL-DH cells, selinexor and INCB057643 acted synergistically to upregulate p53 expression and activate its targeted pathways.

This study demonstrated that the BET inhibitor INCB057643 synergized with XPO1 inhibitors, either selinexor or eltanexor, to increase TP53-mutated and TP53 wild-type cell death in vitro and in vivo. Downregulation in MYC expression was the possible mechanism underlying the synergy of this drug combination in HGBCL-DH cells independent of the TP53 mutation status. Induction of DNA damage and disruption of the DDR were involved in the therapeutic cooperation of these drugs in TP53-mutated cell death. INCB057643 and selinexor synergistically upregulated p53 expression and activated its targeted pathways in TP53 wild-type HGBCL-DH cells. In conclusion, our findings might provide a potential promising combination therapeutic regimen for the hard-to-treat HGBCL-DH. Despite the attractive preclinical results, clinical studies are mandatory to further validate the conclusion.

### Supplementary Information

Supplementary Figures.

Supplementary Information 1.

## Data Availability

All data were included in this manuscript and could be obtained from the corresponding authors via email.
