# Supplementary material for: The synergy of the XPO1 inhibitors combined with the BET inhibitor INCB057643 in high-grade B-cell lymphoma via downregulation of MYC expression
Source: Sci Rep. 2023 Oct 29;13:18554. doi: 10.1038/s41598-023-45721-z (PMC10613613; doi:10.1038/s41598-023-45721-z)
Supplement: Supplementary file 1 — Supplementary Figures. [file 41598_2023_45721_MOESM1_ESM.pdf]

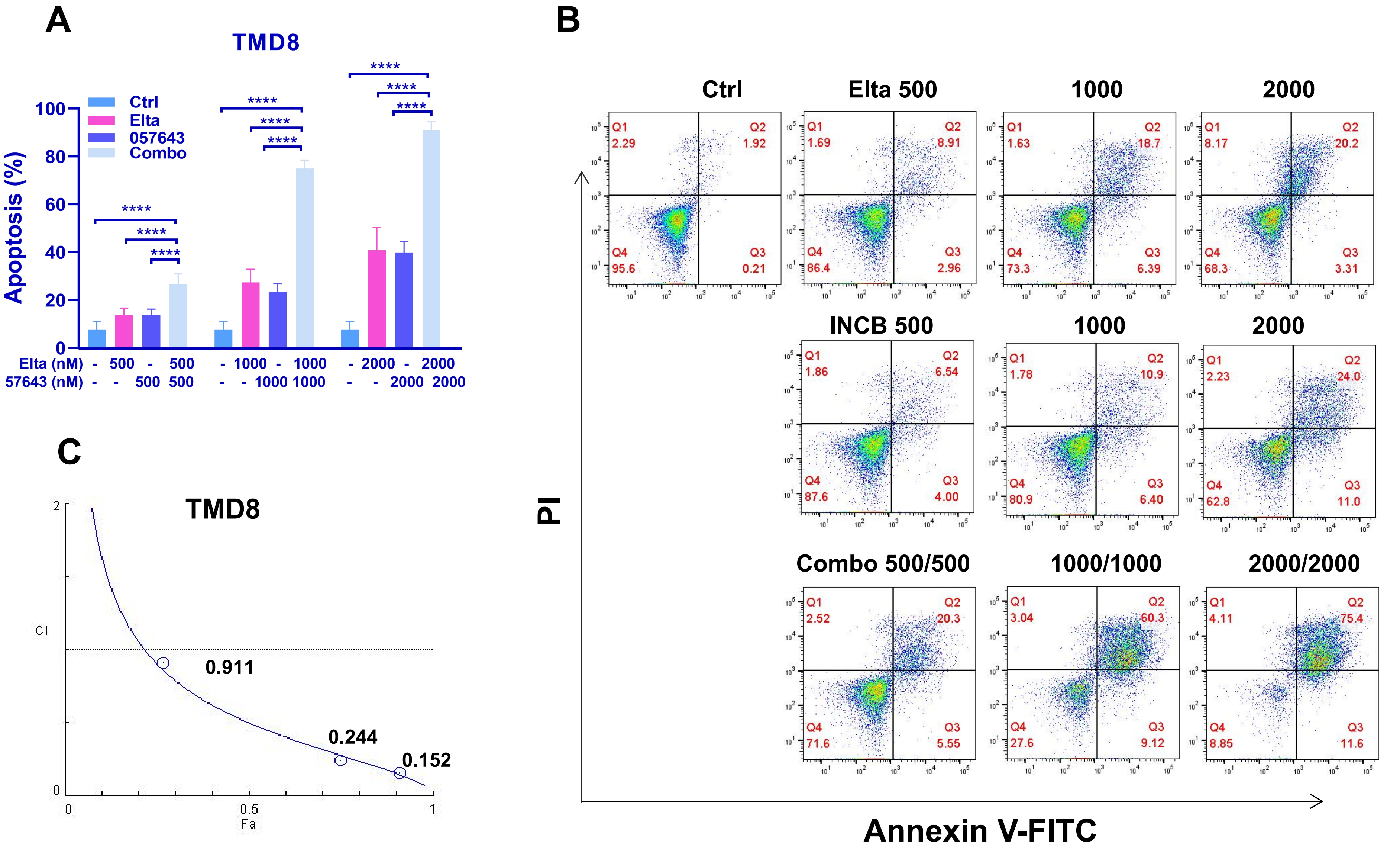

Supplementary Figure S1. INCB057643 synergizes with eltanexor to promote apoptosis in HGBCL-DH cells with TP53 mutations. (A) The synergistic proapoptotic effects of INCB057643 in combination with selinexor in TMD8 cells. **(B)** Representative flow plots of TMD8 cells treated as described in **(A)**. **(C)** Analysis of the CI values based on the apoptosis results of TMD8 cells treated as described in **(A)**.

Supplementary Figure S2

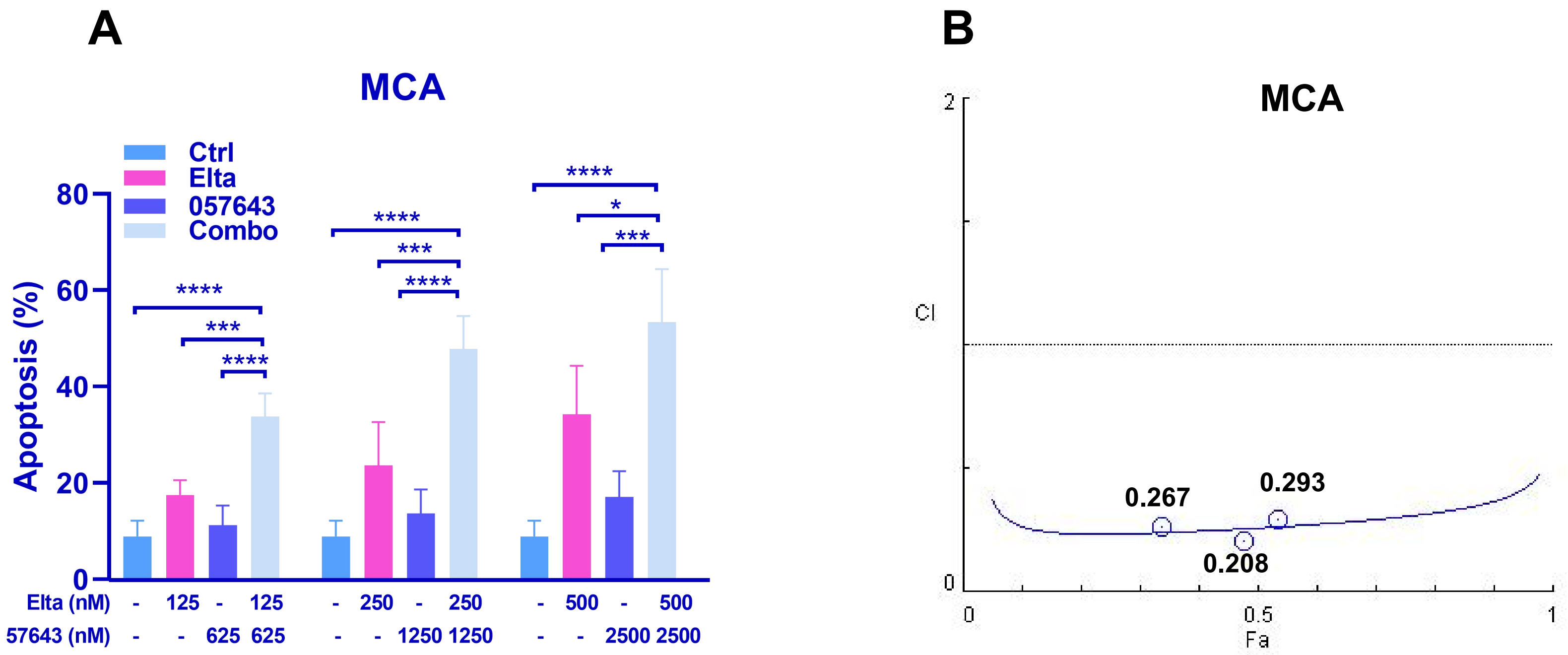

Supplementary Figure S2. The synergistic proapoptotic activity of INCB057643 combined with eltanexor in HGBCL-DH cells without TP53 mutations. (A) INCB057643 and eltanexor were synergisitc to increase the apoptotic proption in MCA cells. **(B)** Analysis of the CI values based on the apoptosis results of MCA cells treated as described in (A).
