# Supplementary material for: The synergy of the XPO1 inhibitors combined with the BET inhibitor INCB057643 in high-grade B-cell lymphoma via downregulation of MYC expression
Source: Sci Rep. 2023 Oct 29;13:18554. doi: 10.1038/s41598-023-45721-z (PMC10613613; doi:10.1038/s41598-023-45721-z)

Protein bands for OCI-LY19 cell line

- **MYC, CHK1, p-CHK1, Rad51**

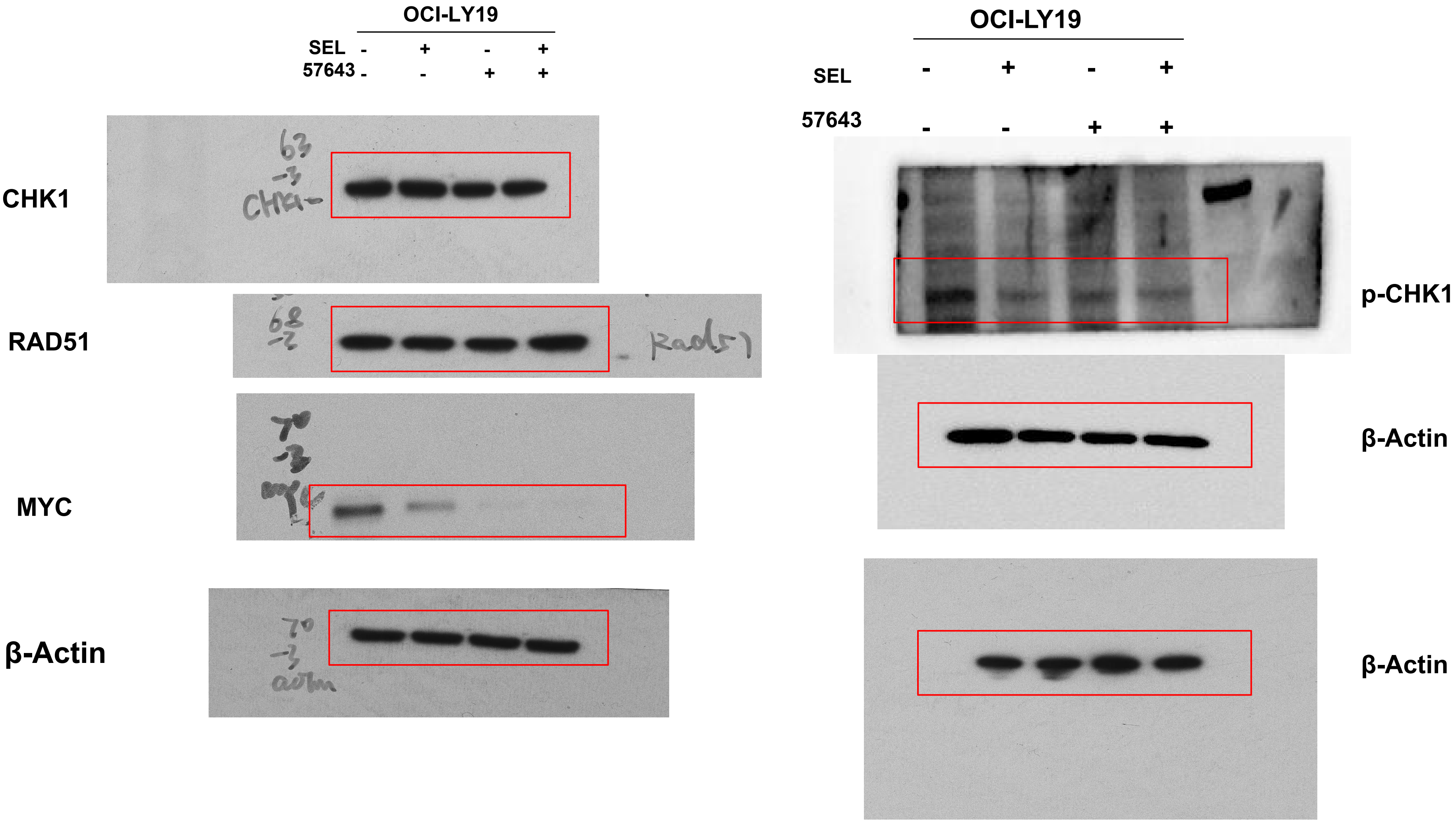

- **CHK2, p-CHK2, r-H2AX**

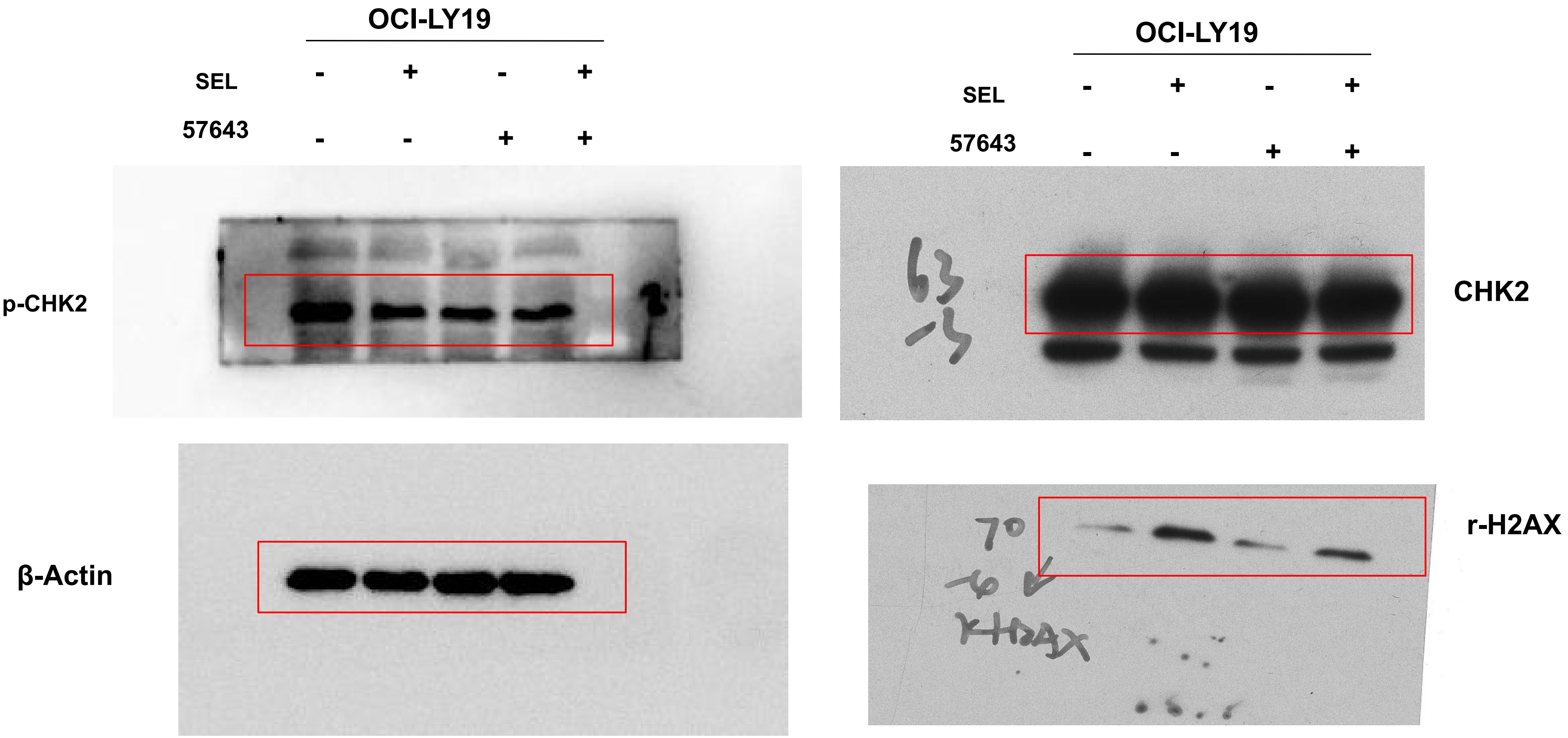

- **p-ATM, p-ATR, p-WEE1**
- **ATR, WEE1**

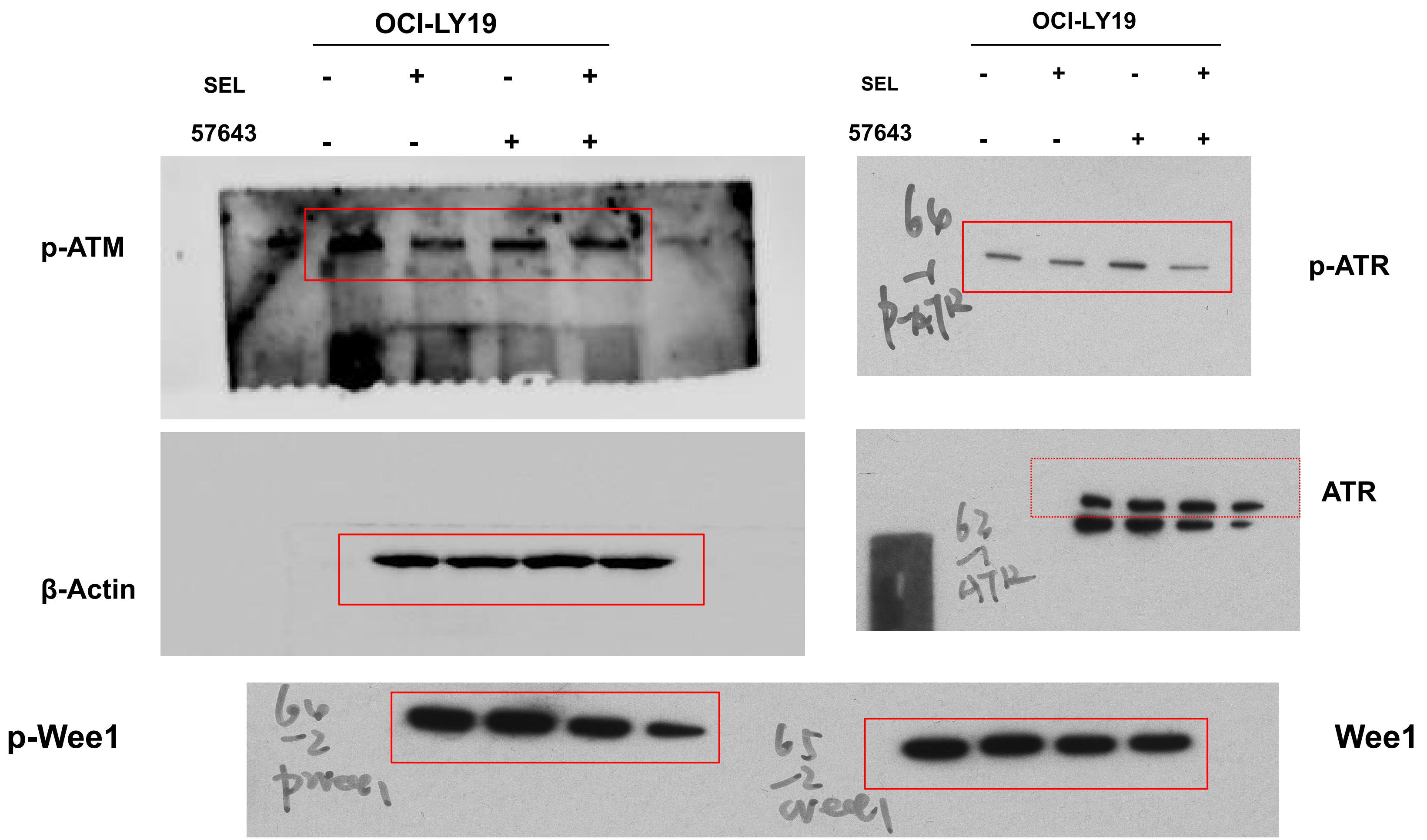

- **P53, MDM2, P21, NOXA, PUMA**
- **BCL-XL, BIM, BCL2, MCL1, Survivin**

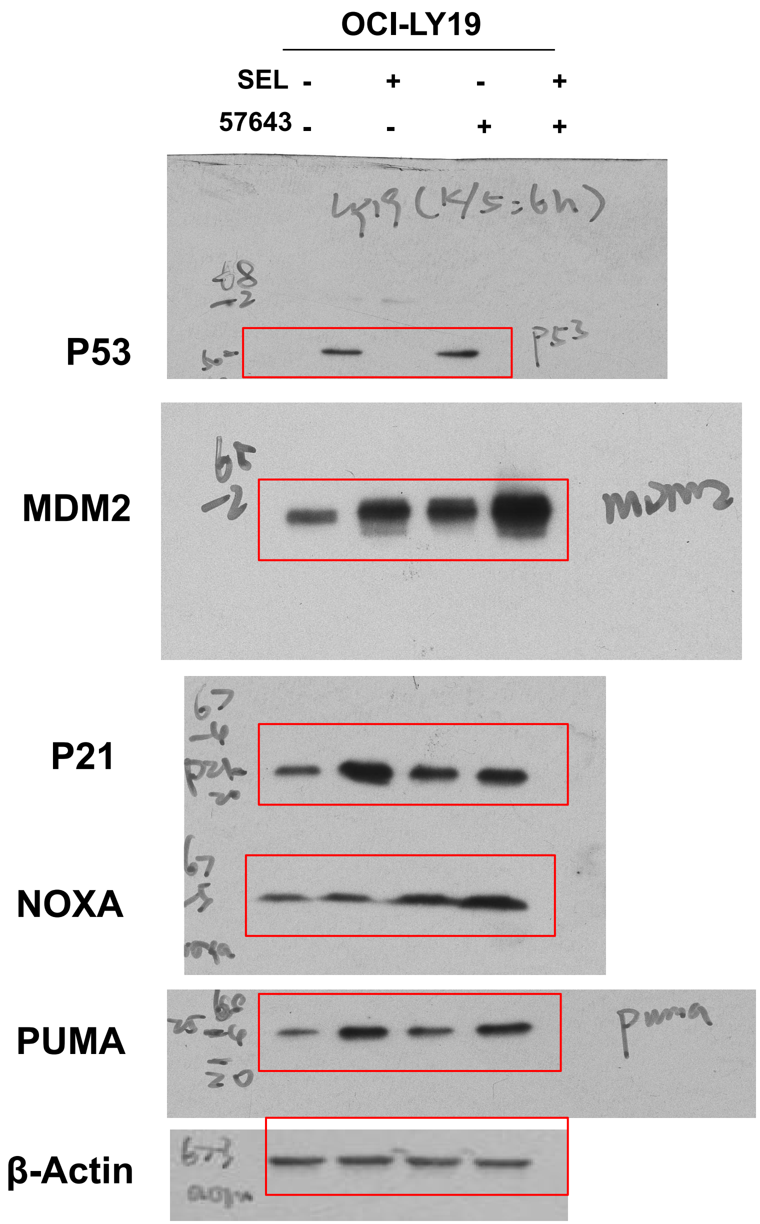

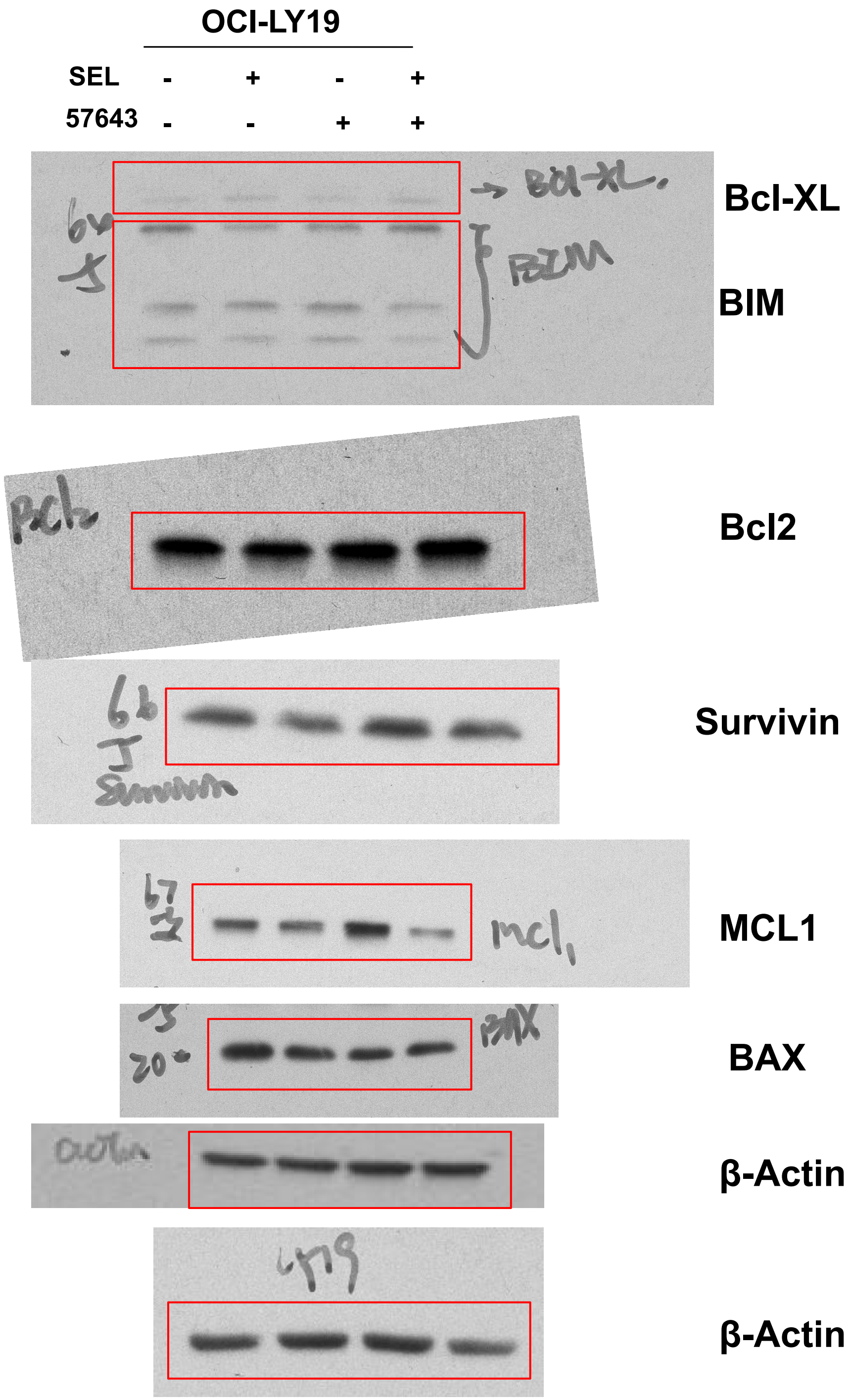

Protein bands for TMD8 cell line

- **ATM, ATR, WEE1, CHK1, CHK2**
- **p-ATM, p-ATR, p-WEE1, p-CHK1, p-CHK2, r-H2AX, Rad 51**

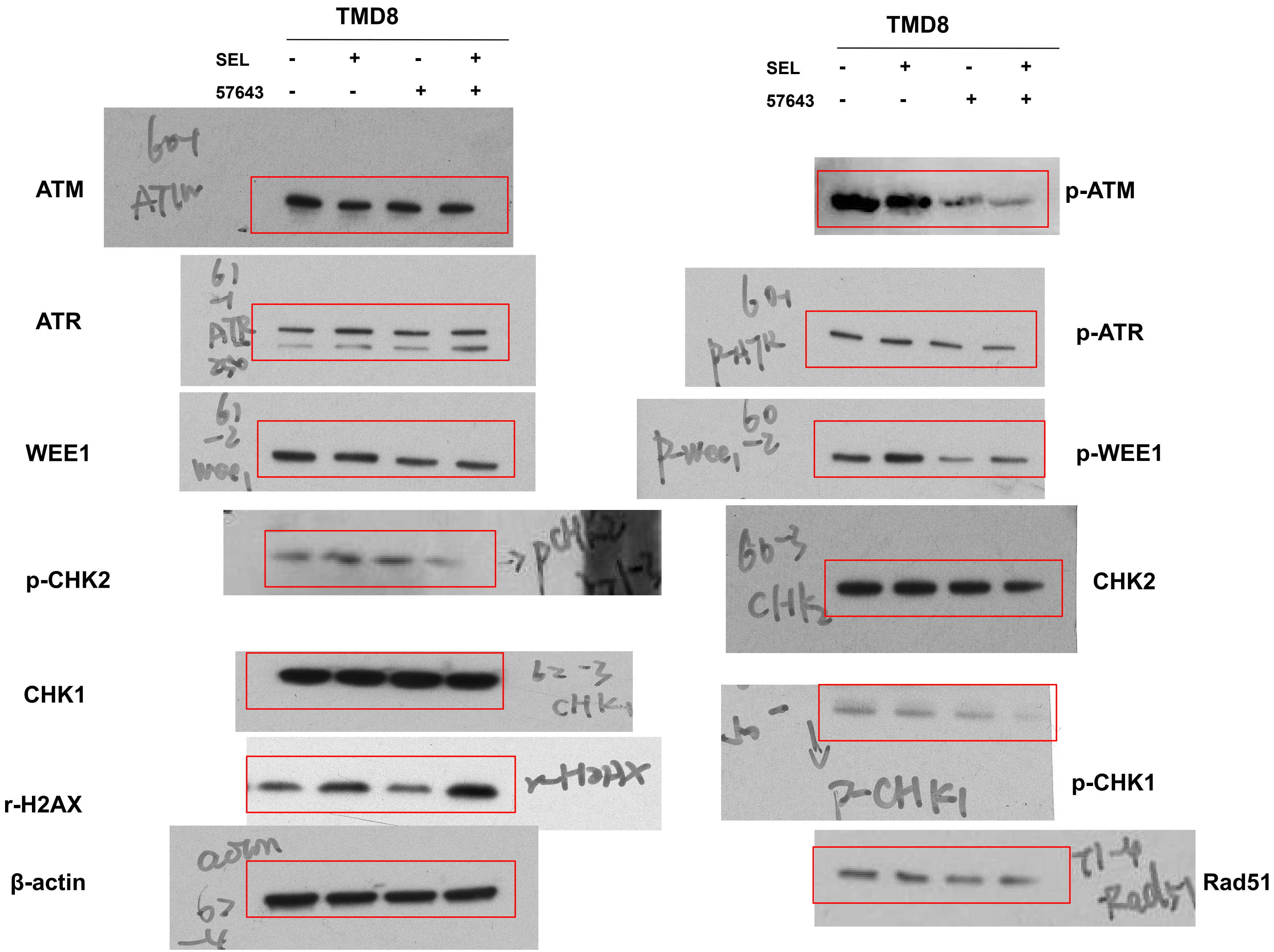

- **MYC, P53, P73, NOXA, PUMA, BAX, BCL2**
- **BIM, BAK, MCL1, Survivin, BCL-XL**

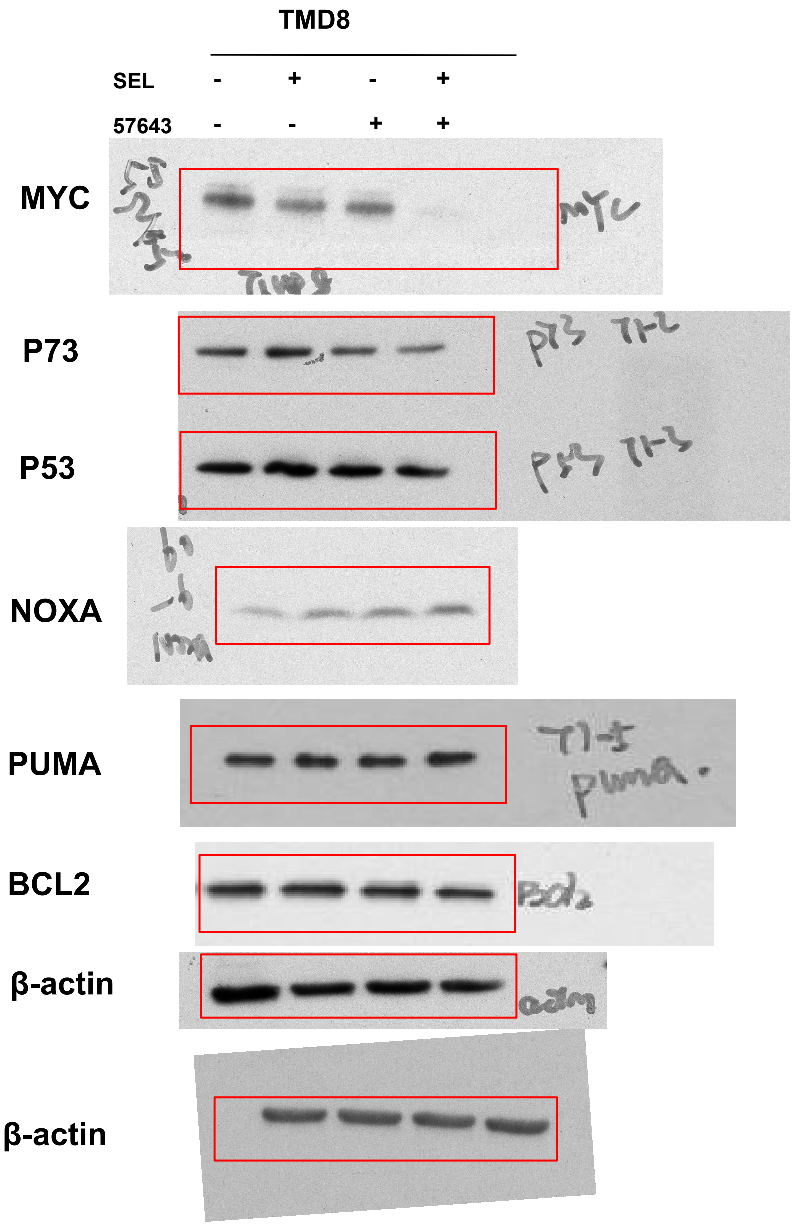

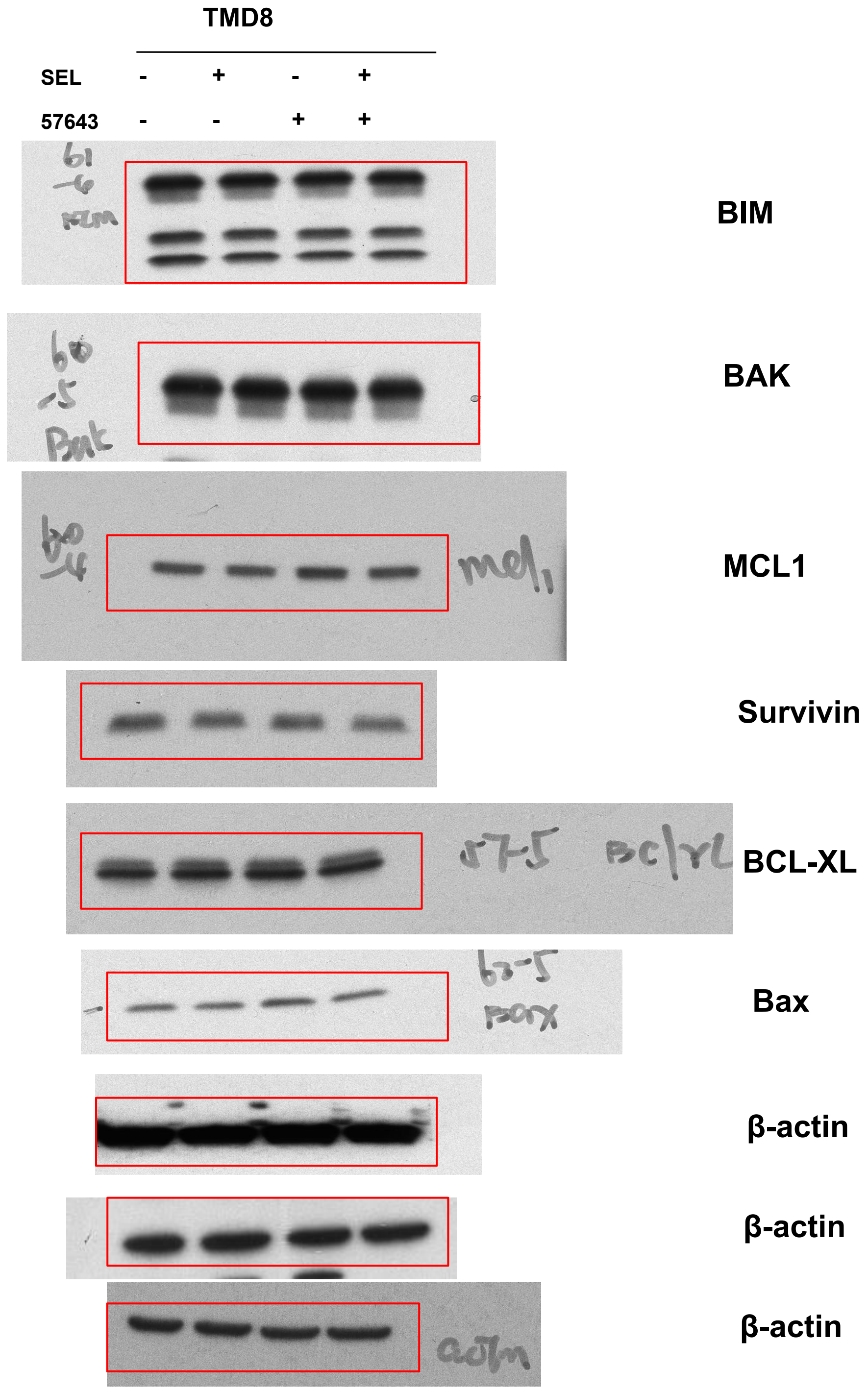

Supplement: Supplementary file 2 — Supplementary Information 1. [file 41598_2023_45721_MOESM2_ESM.pdf]
